# 1,3-Dimeth­oxy-2,3-dihydro-1*H*-isoindole-2-carbothio­amide

**DOI:** 10.1107/S1600536808040075

**Published:** 2008-12-06

**Authors:** Bushra Maliha, Muhammad Ilyas Tariq, M. Nawaz Tahir, Ishtiaq Hussain, Muhammad Ali

**Affiliations:** a Institute of Chemistry, University of the Punjab, Lahore-54590, Pakistan; b Department of Chemistry, University of Sargodha, Sargodha, Pakistan; c Department of Physics, University of Sargodha, Sargodha, Pakistan; d Department of Chemistry, University of Sargodha, Sargodha, Pakistan

## Abstract

In the mol­ecule of the title compound, C_11_H_14_N_2_O_2_S, the five-membered ring adopts an envelope conformation and an intramolecular N—H⋯O hydrogen bond occurs. Intra­molecular N—H⋯O, C—H⋯S and C—H⋯N hydrogen bonds result in the formation of two five- and one six-membered rings, having twisted conformations. In the crystal structure, inter­molecular N—H⋯O, N—H⋯S and C—H⋯S hydrogen bonds link the mol­ecules, forming polymeric sheets. The π–π contacts between the isoindole ring systems, [centroid–centroid distances = 3.5883 (8) and 4.0619 (8) Å] may further stabilize the structure. A C—H⋯π inter­actions also occur.

## Related literature

For general background to isoindoles and their derivatives, see: Mancilla *et al.* (2007 ▶); Toru *et al.* (1986 ▶). For related structures, see: Maliha *et al.* (2007 ▶); Maliha, Hussain *et al.* (2008 ▶); Maliha, Tariq *et al.* (2008 ▶). For bond-length data, see: Allen *et al.* (1987 ▶). 
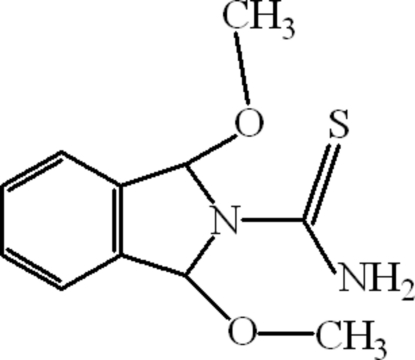


## Experimental

### 

#### Crystal data

C_11_H_14_N_2_O_2_S
*M* *_r_* = 238.30
Monoclinic, 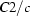

*a* = 15.4577 (8) Å
*b* = 8.6455 (5) Å
*c* = 18.2184 (10) Å
β = 107.322 (2)°
*V* = 2324.3 (2) Å^3^
*Z* = 8
Mo *K*α radiation
μ = 0.27 mm^−1^
*T* = 100 (2) K
0.20 × 0.16 × 0.12 mm

#### Data collection

Bruker Kappa APEXII CCD diffractometer
Absorption correction: multi-scan (*SADABS*; Bruker, 2005 ▶) *T*_min_ = 0.945, *T*_max_ = 0.969
18083 measured reflections
2894 independent reflections
2471 reflections with *I* > 2σ(*I*)
*R*_int_ = 0.032

#### Refinement

*R*[*F* ^2^ > 2σ(*F* ^2^)] = 0.032
*wR*(*F* ^2^) = 0.110
*S* = 1.01
2894 reflections
159 parameters
H atoms treated by a mixture of independent and constrained refinement
Δρ_max_ = 0.44 e Å^−3^
Δρ_min_ = −0.37 e Å^−3^

### 

Data collection: *APEX2* (Bruker, 2007 ▶); cell refinement: *SAINT* (Bruker, 2007 ▶); data reduction: *SAINT*; program(s) used to solve structure: *SHELXS97* (Sheldrick, 2008 ▶); program(s) used to refine structure: *SHELXL97* (Sheldrick, 2008 ▶); molecular graphics: *ORTEP-3 for Windows* (Farrugia, 1997 ▶) and *PLATON* (Spek, 2003 ▶); software used to prepare material for publication: *WinGX* publication routines (Farrugia, 1999 ▶) and *PLATON*.

## Supplementary Material

Crystal structure: contains datablocks text, I. DOI: 10.1107/S1600536808040075/hk2586sup1.cif

Structure factors: contains datablocks I. DOI: 10.1107/S1600536808040075/hk2586Isup2.hkl

Additional supplementary materials:  crystallographic information; 3D view; checkCIF report

## Figures and Tables

**Table 1:** Hydrogen-bond geometry (Å, °)

*D*—H⋯*A*	*D*—H	H⋯*A*	*D*⋯*A*	*D*—H⋯*A*
N2—H1*N*⋯O1^i^	0.891 (17)	2.087 (17)	2.9738 (14)	172.9 (15)
N2—H2*N*⋯O1	0.826 (17)	2.413 (17)	2.9867 (14)	127.3 (14)
N2—H2*N*⋯S1^ii^	0.826 (17)	2.646 (17)	3.2857 (11)	135.4 (15)
C3—H3⋯S1^iii^	0.95	2.79	3.7362 (13)	178
C1—H1⋯*CgB*^iv^	0.98	2.500 (17)	3.4059 (13)	155.1 (1)

Symmetry codes: (i) 
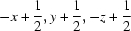
; (ii) 
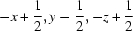
; (iii) 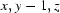; (iv) 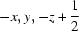. *CgB* is the centroids of the N1/C1/C2/C7/C8 ring.

**Fig. 1.**
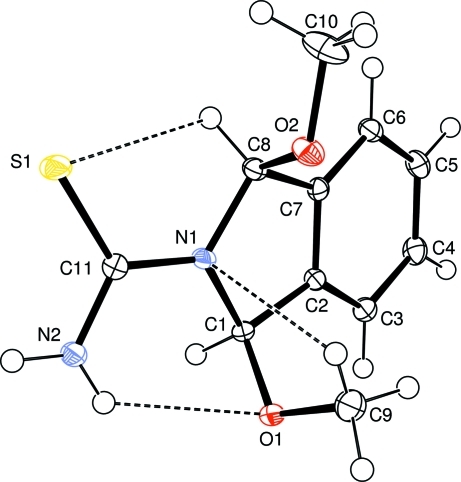
The molecular structure of the title molecule, with the atom-numbering scheme. Displacement ellipsoids are drawn at the 50% probability level. Hydrogen bonds are shown as dashed lines.

**Fig. 2.**
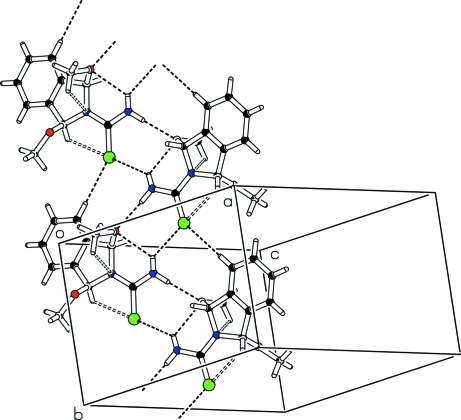
A partial packing diagram of the title compound. Hydrogen bonds are shown as dashed lines.

## supplementary crystallographic information

### Comment

Isoindoles and their derivatives are of great pharmaceutical importance (Mancilla *et al.*,  2007). Certain derivatives of isoindoles have shown a wide range of herbicidal activities (Toru *et al.*,  1986). The title compound is in continuation of the syntheses of isoindoles along with their derivatives and characterizations with the help of X-ray crystallography (Maliha *et al.*,  2007; Maliha, Hussain *et al.*, 2008; Maliha, Tariq *et al.*, 2008).

In the molecule of title compound (Fig 1), the bond lengths (Allen *et al.*,  1987) and angles are within normal ranges. Ring A (C2-C7) is, of course, planar, while the five-membered ring B (N1/C1/C2/C7/C8) adopts envelope conformation with C8 atom displaced by 0.141 (3) Å from the plane of the other ring atoms. The intramolecular N-H···O, C-H···S and C-H···N hydrogen bonds (Table 1) result in the formation of two five- and one six-membered rings: C (N1/S1/C8/C11/H8), D (O1/N1/N2/C1/C11/H2N) and E (O1/N1/C1/C9/H9C), respectively, having twisted conformations.

In the crystal structure, intermolecular N-H···O, N-H···S and C-H···S hydrogen bonds (Table 1) link the molecules to form polymeric sheets, in which the orientations of O—CH_3_ groups cause to the R and S-configurations at the carbon atoms, C1 and C8, respectively. The behaviour of the O—CH_3_ groups are not identical, because only opposite of S-atom is involved in intramolecular H-bonding. The π-π contacts between the isoindole ring systems, CgB—CgB^i^ and CgB—CgA^i^ [symmetry code: (i) -x, y, 1/2 - z, where CgA and CgB are centroids of the rings A (C2-C7) and B (N1/C1/C2/C7/C8)] may further stabilize the structure, with centroid-centroid distances of 3.5883 (8) Å and 4.0619 (8) Å. There also exist two C–H···π interactions (Table 1).

### Experimental

For the preparation of the title conpound, ortho-phthaldehyde (1.34 g, 200 mmol) and thiourea (0.76 g, 200 mmol) were added to distilled water (250 ml), and aqueous NaOH (5 ml, 5%) was added dropwise with constant stirring. After 3 h, a colorless precipitate was obtained, which was washed with hexane, ethanol, acetone and methanol, respectively. Then, it was further refluxed in methanol for 2 h, and left to stand overnight. The deep red tiny crystals settled down, which were washed with ether, hexane and cold methanol, respectively. Crystals suitable for X-ray analysis were obtained from a solution of acetone/methanol mixture by slow evaporation at room temperature.

### Refinement

H1, H8 (for CH) and H1N, H2N (for NH_2_) atoms were located in difference syntheses and refined [C-H = 0.972 (17) and 0.979 (16) Å, N-H = 0.891 (17) and 0.826 (17) Å; U_iso_(H) = 1.2U_eq_(C,N). The remaining H atoms were positioned geometrically, with C-H = 0.95 and 0.98 Å for aromatic and methyl H, respectively, and constrained to ride on their parent atoms with U_iso_(H) = xU_eq_(C), where x = 1.5 for methyl H and x = 1.2 for aromatic H atoms.

### Crystal data

**Table:**

C_11_H_14_N_2_O_2_S	*F*(000) = 1008
*M**_r_* = 238.30	*D*_x_ = 1.362 Mg m^−^^3^
Monoclinic, *C*2/*c*	Mo *K*α radiation, λ = 0.71073 Å
Hall symbol: -C 2yc	Cell parameters from 1295 reflections
*a* = 15.4577 (8) Å	θ = 2.3–28.3°
*b* = 8.6455 (5) Å	µ = 0.27 mm^−^^1^
*c* = 18.2184 (10) Å	*T* = 100 K
β = 107.322 (2)°	Prismatic, red
*V* = 2324.3 (2) Å^3^	0.20 × 0.16 × 0.12 mm
*Z* = 8	

### Data collection

**Table:**

Bruker Kappa APEXII CCD diffractometer	2894 independent reflections
Radiation source: fine-focus sealed tube	2471 reflections with *I* > 2σ(*I*)
graphite	*R*_int_ = 0.032
Detector resolution: 7.40 pixels mm^-1^	θ_max_ = 28.3°, θ_min_ = 2.3°
ω scans	*h* = −20→20
Absorption correction: multi-scan (*SADABS*; Bruker, 2005)	*k* = −11→11
*T*_min_ = 0.945, *T*_max_ = 0.969	*l* = −24→23
18083 measured reflections	

### Refinement

**Table:**

Refinement on *F*^2^	Primary atom site location: structure-invariant direct methods
Least-squares matrix: full	Secondary atom site location: difference Fourier map
*R*[*F*^2^ > 2σ(*F*^2^)] = 0.032	Hydrogen site location: inferred from neighbouring sites
*wR*(*F*^2^) = 0.110	H atoms treated by a mixture of independent and constrained refinement
*S* = 1.01	*w* = 1/[σ^2^(*F*_o_^2^) + (0.0799*P*)^2^ + 0.660*P*] where *P* = (*F*_o_^2^ + 2*F*_c_^2^)/3
2894 reflections	(Δ/σ)_max_ = 0.002
159 parameters	Δρ_max_ = 0.44 e Å^−^^3^
0 restraints	Δρ_min_ = −0.37 e Å^−^^3^

### Special details

**Table:**

Geometry. Bond distances, angles *etc*. have been calculated using the rounded fractional coordinates. All su's are estimated from the variances of the (full) variance-covariance matrix. The cell e.s.d.'s are taken into account in the estimation of distances, angles and torsion angles
Refinement. Refinement of *F*^2^ against ALL reflections. The weighted *R*-factor *wR* and goodness of fit *S* are based on *F*^2^, conventional *R*-factors *R* are based on *F*, with *F* set to zero for negative *F*^2^. The threshold expression of *F*^2^ > σ(*F*^2^) is used only for calculating *R*-factors(gt) *etc*. and is not relevant to the choice of reflections for refinement. *R*-factors based on *F*^2^ are statistically about twice as large as those based on *F*, and *R*- factors based on ALL data will be even larger.

### Fractional atomic coordinates and isotropic or equivalent isotropic displacement parameters (Å^2^)

**Table:**

	*x*	*y*	*z*	*U*_iso_*/*U*_eq_	
S1	0.11517 (2)	0.49877 (3)	0.21266 (2)	0.0204 (1)	
O1	0.14785 (6)	−0.02891 (10)	0.15401 (5)	0.0138 (2)	
O2	−0.01835 (6)	0.30468 (10)	0.05803 (5)	0.0171 (2)	
N1	0.07220 (7)	0.20405 (11)	0.17484 (6)	0.0120 (3)	
N2	0.21825 (7)	0.25081 (12)	0.24928 (6)	0.0148 (3)	
C1	0.08273 (8)	0.03577 (13)	0.18556 (7)	0.0110 (3)	
C2	−0.01336 (8)	−0.02006 (14)	0.15260 (7)	0.0117 (3)	
C3	−0.04573 (8)	−0.17035 (14)	0.15157 (7)	0.0154 (3)	
C4	−0.13922 (9)	−0.19328 (15)	0.12533 (8)	0.0174 (3)	
C5	−0.19841 (9)	−0.06902 (15)	0.10138 (7)	0.0167 (3)	
C6	−0.16516 (8)	0.08113 (14)	0.10175 (7)	0.0141 (3)	
C7	−0.07174 (8)	0.10380 (13)	0.12693 (7)	0.0119 (3)	
C8	−0.01925 (8)	0.25238 (13)	0.13215 (7)	0.0128 (3)	
C9	0.12812 (10)	−0.01421 (17)	0.07232 (8)	0.0221 (4)	
C10	−0.08727 (10)	0.41497 (17)	0.02569 (9)	0.0275 (4)	
C11	0.13705 (8)	0.30768 (14)	0.21217 (7)	0.0125 (3)	
H1	0.1091 (11)	0.0123 (16)	0.2398 (10)	0.0132*	
H1N	0.2602 (11)	0.318 (2)	0.2748 (9)	0.0178*	
H2N	0.2325 (11)	0.160 (2)	0.2447 (9)	0.0178*	
H3	−0.00537	−0.25487	0.16825	0.0184*	
H4	−0.16291	−0.29503	0.12374	0.0208*	
H5	−0.26194	−0.08673	0.08467	0.0201*	
H6	−0.20533	0.16594	0.08519	0.0169*	
H8	−0.0393 (10)	0.3344 (18)	0.1604 (9)	0.0153*	
H9A	0.07524	−0.07779	0.04676	0.0265*	
H9B	0.18042	−0.04909	0.05681	0.0265*	
H9C	0.11520	0.09429	0.05753	0.0265*	
H10A	−0.14679	0.36807	0.01925	0.0330*	
H10B	−0.08290	0.44837	−0.02449	0.0330*	
H10C	−0.07967	0.50456	0.05998	0.0330*	

### Atomic displacement parameters (Å^2^)

**Table:**

	*U*^11^	*U*^22^	*U*^33^	*U*^12^	*U*^13^	*U*^23^
S1	0.0134 (2)	0.0095 (2)	0.0347 (2)	0.0004 (1)	0.0018 (2)	−0.0017 (1)
O1	0.0126 (4)	0.0154 (4)	0.0131 (4)	0.0039 (3)	0.0032 (3)	−0.0015 (3)
O2	0.0180 (4)	0.0163 (4)	0.0155 (4)	0.0019 (3)	0.0025 (4)	0.0062 (3)
N1	0.0103 (5)	0.0095 (4)	0.0144 (5)	0.0014 (3)	0.0010 (4)	0.0007 (4)
N2	0.0121 (5)	0.0107 (5)	0.0195 (5)	0.0001 (4)	0.0013 (4)	−0.0006 (4)
C1	0.0115 (5)	0.0089 (5)	0.0119 (5)	0.0016 (4)	0.0024 (4)	0.0000 (4)
C2	0.0105 (5)	0.0133 (5)	0.0109 (5)	0.0006 (4)	0.0025 (4)	−0.0007 (4)
C3	0.0161 (6)	0.0124 (5)	0.0162 (6)	0.0005 (4)	0.0026 (5)	−0.0008 (4)
C4	0.0182 (6)	0.0145 (6)	0.0184 (6)	−0.0043 (4)	0.0040 (5)	−0.0014 (5)
C5	0.0123 (5)	0.0208 (6)	0.0160 (6)	−0.0035 (5)	0.0027 (5)	−0.0001 (5)
C6	0.0126 (5)	0.0160 (6)	0.0130 (6)	0.0016 (4)	0.0027 (4)	0.0011 (4)
C7	0.0134 (5)	0.0121 (5)	0.0098 (5)	0.0006 (4)	0.0030 (4)	−0.0004 (4)
C8	0.0113 (5)	0.0116 (5)	0.0138 (5)	0.0013 (4)	0.0013 (4)	0.0011 (4)
C9	0.0224 (7)	0.0315 (8)	0.0134 (6)	0.0055 (5)	0.0069 (5)	−0.0018 (5)
C10	0.0194 (6)	0.0282 (7)	0.0313 (8)	0.0045 (5)	0.0021 (6)	0.0163 (6)
C11	0.0127 (5)	0.0127 (5)	0.0126 (5)	−0.0006 (4)	0.0046 (4)	0.0003 (4)

### Geometric parameters (Å, °)

**Table:**

S1—C11	1.6869 (12)	C5—C6	1.3954 (18)
O1—C1	1.4145 (16)	C6—C7	1.3927 (18)
O1—C9	1.4331 (16)	C7—C8	1.5072 (17)
O2—C8	1.4280 (15)	C1—H1	0.972 (17)
O2—C10	1.4204 (18)	C3—H3	0.9500
N1—C1	1.4705 (15)	C4—H4	0.9500
N1—C8	1.4571 (17)	C5—H5	0.9500
N1—C11	1.3653 (16)	C6—H6	0.9500
N2—C11	1.3300 (17)	C8—H8	0.979 (16)
N2—H1N	0.891 (17)	C9—H9A	0.9800
N2—H2N	0.826 (17)	C9—H9B	0.9800
C1—C2	1.5062 (18)	C9—H9C	0.9800
C2—C7	1.3887 (17)	C10—H10A	0.9800
C2—C3	1.3905 (17)	C10—H10B	0.9800
C3—C4	1.3946 (19)	C10—H10C	0.9800
C4—C5	1.3942 (19)		
			
S1···O2	3.3984 (10)	C6···H10A	2.9600
S1···N2^i^	3.2857 (11)	C6···H1^vi^	2.819 (17)
S1···H3^ii^	2.7900	C6···H9B^iv^	2.8400
S1···H8	2.697 (16)	C7···H1^vi^	2.773 (17)
S1···H2N^i^	2.646 (17)	C7···H10A	3.0100
O1···N2	2.9867 (14)	C10···H10B^v^	2.8900
O1···N2^iii^	2.9738 (14)	C10···H5^vii^	2.9800
O2···S1	3.3984 (10)	H1···N2	2.637 (16)
O1···H1N^iii^	2.087 (17)	H1···H2N	2.28 (2)
O1···H2N	2.413 (17)	H1···C2^vi^	2.800 (18)
O2···H9C	2.7500	H1···C3^vi^	2.920 (17)
O2···H9A^iv^	2.7000	H1···C4^vi^	2.955 (16)
O2···H10B^v^	2.8200	H1···C5^vi^	2.897 (17)
N2···C6^vi^	3.3938 (16)	H1···C6^vi^	2.819 (17)
N2···O1	2.9867 (14)	H1···C7^vi^	2.773 (17)
N2···O1^i^	2.9738 (14)	H1N···O1^i^	2.087 (17)
N2···S1^iii^	3.2857 (11)	H1N···C1^i^	2.986 (17)
N1···H9C	2.6000	H2N···O1	2.413 (17)
N2···H1	2.637 (16)	H2N···C1	2.488 (17)
C1···C2^vi^	3.4582 (18)	H2N···H1	2.28 (2)
C1···C7^vi^	3.5200 (17)	H2N···S1^iii^	2.646 (17)
C2···C2^vi^	3.4486 (17)	H3···S1^viii^	2.7900
C2···C1^vi^	3.4582 (18)	H5···C10^vii^	2.9800
C3···C3^vi^	3.4413 (17)	H6···H10A	2.4400
C6···N2^vi^	3.3938 (16)	H6···H10A^vii^	2.5200
C6···C9^iv^	3.4352 (19)	H8···S1	2.697 (16)
C6···C10	3.563 (2)	H8···H10C	2.2800
C7···C1^vi^	3.5200 (17)	H9A···C2	2.7200
C7···C9^iv^	3.5571 (19)	H9A···O2^iv^	2.7000
C9···C7^iv^	3.5571 (19)	H9B···C6^iv^	2.8400
C9···C6^iv^	3.4352 (19)	H9C···O2	2.7500
C10···C10^v^	3.438 (2)	H9C···N1	2.6000
C10···C6	3.563 (2)	H10A···C6	2.9600
C1···H1N^iii^	2.986 (17)	H10A···C7	3.0100
C1···H2N	2.488 (17)	H10A···H6	2.4400
C2···H9A	2.7200	H10A···H6^vii^	2.5200
C2···H1^vi^	2.800 (18)	H10B···O2^v^	2.8200
C3···H1^vi^	2.920 (17)	H10B···C10^v^	2.8900
C4···H1^vi^	2.955 (16)	H10C···H8	2.2800
C5···H1^vi^	2.897 (17)		
			
C1—O1—C9	115.45 (10)	O1—C1—H1	101.4 (10)
C8—O2—C10	112.80 (10)	N1—C1—H1	109.8 (8)
C1—N1—C8	113.96 (10)	C2—C1—H1	113.8 (10)
C1—N1—C11	123.18 (10)	C2—C3—H3	121.00
C8—N1—C11	121.97 (10)	C4—C3—H3	121.00
H1N—N2—H2N	119.8 (16)	C3—C4—H4	120.00
C11—N2—H1N	117.0 (11)	C5—C4—H4	120.00
C11—N2—H2N	122.7 (12)	C4—C5—H5	120.00
O1—C1—N1	113.65 (10)	C6—C5—H5	120.00
O1—C1—C2	116.61 (10)	C5—C6—H6	121.00
N1—C1—C2	101.95 (10)	C7—C6—H6	121.00
C1—C2—C3	127.81 (11)	O2—C8—H8	111.2 (9)
C3—C2—C7	121.39 (12)	N1—C8—H8	109.6 (9)
C1—C2—C7	110.61 (10)	C7—C8—H8	113.7 (9)
C2—C3—C4	118.00 (12)	O1—C9—H9A	109.00
C3—C4—C5	120.95 (12)	O1—C9—H9B	109.00
C4—C5—C6	120.59 (13)	O1—C9—H9C	109.00
C5—C6—C7	118.48 (11)	H9A—C9—H9B	109.00
C2—C7—C6	120.56 (11)	H9A—C9—H9C	109.00
C2—C7—C8	110.63 (11)	H9B—C9—H9C	109.00
C6—C7—C8	128.80 (11)	O2—C10—H10A	109.00
N1—C8—C7	101.99 (9)	O2—C10—H10B	109.00
O2—C8—N1	108.33 (10)	O2—C10—H10C	109.00
O2—C8—C7	111.52 (10)	H10A—C10—H10B	109.00
S1—C11—N1	121.78 (10)	H10A—C10—H10C	109.00
S1—C11—N2	121.31 (10)	H10B—C10—H10C	109.00
N1—C11—N2	116.90 (11)		
			
C9—O1—C1—N1	−62.73 (14)	N1—C1—C2—C3	−175.58 (12)
C9—O1—C1—C2	55.45 (14)	N1—C1—C2—C7	−0.62 (13)
C10—O2—C8—N1	−153.14 (10)	C1—C2—C3—C4	173.19 (12)
C10—O2—C8—C7	95.39 (12)	C7—C2—C3—C4	−1.28 (19)
C8—N1—C1—O1	120.77 (11)	C1—C2—C7—C6	−173.11 (11)
C8—N1—C1—C2	−5.57 (13)	C1—C2—C7—C8	6.27 (14)
C11—N1—C1—O1	−69.90 (15)	C3—C2—C7—C6	2.22 (19)
C11—N1—C1—C2	163.77 (11)	C3—C2—C7—C8	−178.40 (11)
C1—N1—C8—O2	−108.78 (11)	C2—C3—C4—C5	−0.45 (19)
C1—N1—C8—C7	8.96 (13)	C3—C4—C5—C6	1.3 (2)
C11—N1—C8—O2	81.74 (13)	C4—C5—C6—C7	−0.33 (19)
C11—N1—C8—C7	−160.52 (11)	C5—C6—C7—C2	−1.37 (18)
C1—N1—C11—S1	−167.45 (9)	C5—C6—C7—C8	179.37 (12)
C1—N1—C11—N2	11.57 (18)	C2—C7—C8—O2	106.35 (12)
C8—N1—C11—S1	1.05 (17)	C2—C7—C8—N1	−9.08 (13)
C8—N1—C11—N2	−179.93 (11)	C6—C7—C8—O2	−74.34 (16)
O1—C1—C2—C3	60.04 (17)	C6—C7—C8—N1	170.24 (12)
O1—C1—C2—C7	−125.00 (11)		

Symmetry codes: (i) −*x*+1/2, *y*+1/2, −*z*+1/2; (ii) *x*, *y*+1, *z*; (iii) −*x*+1/2, *y*−1/2, −*z*+1/2; (iv) −*x*, −*y*, −*z*; (v) −*x*, −*y*+1, −*z*; (vi) −*x*, *y*, −*z*+1/2; (vii) −*x*−1/2, −*y*+1/2, −*z*; (viii) *x*, *y*−1, *z*.

### Hydrogen-bond geometry (Å, °)

**Table:**

*D*—H···*A*	*D*—H	H···*A*	*D*···*A*	*D*—H···*A*
N2—H1N···O1^i^	0.891 (17)	2.087 (17)	2.9738 (14)	172.9 (15)
N2—H2N···O1	0.826 (17)	2.413 (17)	2.9867 (14)	127.3 (14)
N2—H2N···S1^iii^	0.826 (17)	2.646 (17)	3.2857 (11)	135.4 (15)
C3—H3···S1^viii^	0.9500	2.7900	3.7362 (13)	178.00
C8—H8···S1	0.979 (16)	2.697 (16)	3.0318 (12)	100.5 (11)
C9—H9C···N1	0.9800	2.6000	2.9593 (18)	102.00
C1—H1···CgB^vi^	0.9800	2.500 (17)	3.4059 (13)	155.1 (1)
C9—H9C···CgA	0.9800	2.74	2.9052 (16)	90

Symmetry codes: (i) −*x*+1/2, *y*+1/2, −*z*+1/2; (iii) −*x*+1/2, *y*−1/2, −*z*+1/2; (viii) *x*, *y*−1, *z*; (vi) −*x*, *y*, −*z*+1/2.
